# Network Meta-Analysis of Different Intravenous Glucocorticoid Regimes for the Treatment of Graves’ Orbitopathy

**DOI:** 10.3389/fphar.2022.785757

**Published:** 2022-04-26

**Authors:** Jun Jia, Jingjian Dong, Lin Deng

**Affiliations:** ^1^ Department of Ophthalmology, The Central Hospital of Wuhan, Tongji Medical College, Huazhong University of Science and Technology, Wuhan, China; ^2^ Department of Endocrinology, The Central Hospital of Wuhan, Tongji Medical College, Huazhong University of Science and Technology, Wuhan, China

**Keywords:** Graves’ disease, Graves’ ophthalmopathy, glucocorticoid, intravenous, network meta-analysis

## Abstract

**Background:** Intravenous glucocorticoid (GC) has been proposed to treat moderately severe Graves’ orbitopathy (GO); however, the optimal regime remains debatable. We therefore performed this network meta-analysis to objectively determine the comparative efficacy and safety of different intravenous GC regimes, including daily, weekly, or monthly intravenous regimes, for the treatment of GO.

**Methods:** We electronically searched Medline (*via* PubMed), EMBASE (*via* OVID), and the Cochrane Central Register of Controlled Trials (CENTRAL) (*via* OVID) to retrieve randomized controlled trials (RCTs) investigating the comparative efficacy and safety of different intravenous GC regimes in GO patients from the inception of each database to March 2021. The latest search was updated in June 2021. The risk of bias of original studies was assessed using the Cochrane risk bias assessment tool. A random-effects Bayesian network meta-analysis was conducted using the Markov chain Monte Carlo (MCMC) simulation. Ranking probabilities of all regimes were calculated to rank all regimes. Statistical analysis was conducted using the ADDIS software.

**Results:** A total of 10 studies involving 593 patients met the selection criteria. Network meta-analysis suggested that the weekly intravenous GC regime (WR) [response: odds ratio (OR), 4.27; 95% creditable interval (CrI); 1.82 to 11.02; clinical activity score change (CASC): standard mean difference (SMD), −0.59; 95% CrI; −1.19 to −0.03) and monthly intravenous regime (MR) (response: OR, 6.32; 95% CrI; 1.25 to 34.96; CASC: SMD, −1.17; 95% CrI; −2.32 to −0.01) were superior to the oral GC (OGC) regime in response and CASC. Meanwhile, pooled results also indicated that the WR was related to the decreased risk of AEs compared with the OGC regime (OR, 0.22; 95% CrI; 0.08–0.62) and daily intravenous GC regime (DR) (OR, 0.19; 95% CrI; 0.03–0.97). Ranking probabilities indicated that the MR and WR have a relatively higher probability of becoming the best option for response, proptosis, and AEs.

**Conclusion:** Based on limited evidence, the WR or MR should be preferentially prescribed to treat patients with moderately severe GO. However, more studies with a large sample size should be conducted to further confirm our findings and compare the WR with the MR.

## Introduction

Graves’ orbitopathy (GO) is one of the common autoimmune disorders and also the most common extrathyroidal manifestations of Graves’ disease (GD) (Bartalena and Tanda, 2009; Barrio-Barrio et al., 2015). GO is closely related to hyperthyroidism, hypothyroidism, or euthyroid, and patients with conditions mentioned above were also found to suffer from GO (Bahn, 2010). GO is a mild and self-limited disease that only requires local treatment rather than intensive therapy (Bartalena et al., 2008); however, for patients with active and moderate-to-severe GO, glucocorticoids have been the most common immunosuppressive agents used in the treatment (Stan et al., 2012; Campi et al., 2022; Rajabi et al., 2022).

In clinical practice, glucocorticoid therapy can be administered orally or intravenously (Kinsell et al., 1953). Among available administration routes, intravenous administration was confirmed to be more effective and safer than other routes, including oral and local routes (Stiebel-Kalish et al., 2009; Gao et al., 2014; Tu et al., 2018; Zhao et al., 2019). Therefore, intravenous glucocorticoid therapy was recommended as the first-line therapy of active moderate-to-severe GO by the European Group on Graves’ Ophthalmopathy (EUGOGO) (Bartalena et al., 2008). However, currently, several questions about intravenous GC therapy have not yet been clearly answered (Zhao et al., 2019).

A previous meta-analysis (Zang et al., 2011) investigated the comparative efficacy and safety of different dose regimes of intravenous GC and suggested that a high-dose regime of intravenous steroids was superior to a lower dose regime in terms of response regardless of single and cumulative doses; however, a high-dose regime was associated with increased risk of occurrence of adverse events (AEs). In actuality, there are three major regimens for intravenous GC therapy, namely, daily (e.g., 0.5 g intravenous methylprednisolone daily for 5 days), weekly (e.g., 0.5 g intravenous methylprednisolone for 6 weeks followed by 0.25 g weekly for 6 weeks), or monthly (e.g., 1.5 g iv intravenous methylprednisolone for 3 months) schemes regardless of the dose (Bartalena et al., 2008). However, the details of the treatment schedule continue to be debatable. Until now, two studies have directly compared daily (DR) with weekly (WR) regimes and one study has directly compared the WR with the monthly regime (MR); however, no study has been performed to compare the DR with the MR. Hence, it is unclear as to which intravenous GC regimes should be preferably selected in clinical practice.

Although conventional pairwise meta-analysis can investigate the comparative efficacy and safety of two comparisons, it does not have the ability to simultaneously investigate the comparative efficacy and safety of more than three comparisons. As an expansion of traditional pairwise meta-analysis, network meta-analysis has been developed to simultaneously investigate more than three comparisons (Mbuagbaw et al., 2017). We, therefore, determined the optimal intravenous regime of GC therapy by introducing a network meta-analysis technique in the present study.

## Methods

We conducted this network meta-analysis according to the recommendations proposed by the Cochrane Collaboration (Higgins and Green, 2011). Meanwhile, we developed the structure of our network meta-analysis and reported all results in line with the Preferred Reporting Items for Systematic Reviews and Meta-Analyses (PRISMA) for Network Meta-Analysis (PRISMA-NMA) checklist (Hutton et al., 2015; Page et al., 2021a; Page et al., 2021b). Our network meta-analysis did not require ethical approval or informed patient consent because all statistical analyses were conducted on the basis of published data.

### Identification of Studies

Two independent reviewers identified eligible studies by electronically searching Medline (using PubMed), EMBASE (using OVID), and the Cochrane Central Register of Controlled Trials (CENTRAL, using OVID) from their inception to March 2021. The latest search was updated in June 2021. Restrictions such as publication language and publication status were not imposed in the literature search. The following terms were used to identify eligible studies: Graves’ ophthalmopathy, glucocorticoid, steroid, methylprednisolone, and random. The initial search strategy was constructed according to the principle of combining medical subject heading (MeSH) with free words. Detailed search strategies are summarized in Supplementary Table S1. Moreover, we checked references of eligible studies and previous meta-analyses to add additional studies. We resolved any disagreements between two reviewers by consulting a third reviewer.

### Selection Criteria

We developed selection criteria including inclusion and exclusion criteria as follows: 1) randomized controlled trials (RCTs) with full texts were considered regardless of language and publication status; 2) adult patients were diagnosed with GO based on the recognized standard; 3) patients were instructed to receive different intravenous GC regimes, patients in the experimental group was assigned to receive the intravenous GC regime, and patients in the control group were assigned to receive oral glucocorticoid (OGC) such as methylprednisolone (MP) and prednisolone (PS); 4)the overall response was defined as the primary outcome, and clinical activity score change (CASC), proptosis, and adverse events (AEs) were defined as the secondary outcomes. CAS is a valid clinical criterion for assessing disease activity in Graves’ orbitopathy (Mourits et al., 1989), and we used the changes in CAS before and after treatment to indicate the therapeutic magnitude of intravenous regimes on disease activity in this meta-analysis.

We excluded any study which met at least one of the following criteria: 1) adequate data are not available for quantitative analysis, 2) duplicate studies with inadequate data or relatively poorer methodological quality, and 3) ineligible design such as narrative review, retrospective studies, or animal study.

### Selection of Studies

Two independent reviewers selected eligible studies according to the selection criteria. We performed the study selection process according to the following three steps: 1) we first removed duplicates by automatically excluding repeated records of the EndNote software; 2) we excluded unrelated records by screening titles and abstracts of retaining records; 3) we retrieved full-texts of records which were in files that store potentially eligible records at the previous stage for further eligibility checking. We resolved any disagreements between two reviewers by consulting a third reviewer.

### Data Extraction

Two independent reviewers extracted essential information using a standard data extraction sheet from each eligible study: the first author’s name, publication year, country of the corresponding author, and sample size accompanied by the number of men, mean age, severity of GO, duration of GO, details of treatment regimes, time of follow-up, outcomes, and details of the risk of bias. We utilized the recognized formula to estimate mean and standard deviation (SD) when a continuous variable was expressed as median and range or interquartile range (IQR) (Wan et al., 2014). We contacted the leading author by email to add additional information. We resolved any disagreements between two reviewers by consulting a third reviewer.

### Risk of Bias Assessment

Two independent reviewers assessed the risk of bias of individual eligible studies using the Cochrane risk bias assessment tool (Higgins et al., 2011) from the following seven items: random sequence generation (selection bias), allocation concealment selection bias, blinding of participants and personnel (performance bias), blinding of outcome assessors (detection bias), incomplete data attrition bias, selective reporting (reporting bias), and other bias (such as inadequate sample size and unfair financial sources). We labeled each item with a low, unclear, or high risk of bias depending on the matching level of actual information of each eligible study and the assessment criteria. The overall methodological quality of the individual study was determined according to the following criteria: 1) a low level was determined if more than one item was rated as high risk of bias; 2) a moderate level was determined if more than one item was rated as unclear risk of bias but no item was rated as high risk of bias; 3) a high level was determined if all items were rated as low risk of bias. We resolved any disagreements between two reviewers by consulting a third reviewer.

### Statistical Analysis

We first performed a conventional pairwise meta-analysis using the random-effects model which considers the fact that variations cannot be avoided in real settings. For dichotomous variables, we calculated the odds ratio (OR) with a 95% confidence interval (CI) to express results. For continuous variables, we calculated the mean difference (MD) or standard MD with 95% CI to express pooled results. We examined heterogeneity across studies for each outcome using Cochran’s Q statistic (based on the chi-square test) (Bowden et al., 2011) and the *I* ^2^ statistic (Higgins and Thompson, 2002). A *p* value of less than 0.1 and an *I* ^2^ value of more than 50% show the presence of substantial heterogeneity across studies. All results of the pairwise meta-analysis were graphically depicted using the Microsoft Excel software.

Following the pairwise meta-analysis, we conducted network meta-analysis using the Aggregate Data Drug Information System software (ADDIS V.1.16.8, Drugis, Groningen, NL), which was developed to calculate all estimates based on the Markov chain Monte Carlo (MCMC) method (Cipriani et al., 2013). Moreover, we utilized the node-split method to perform inconsistency tests when a closed loop was available (Dias et al., 2010), and *p* < 0.05 suggested the presence of inconsistency between direct and indirect effects (Albert and Makowski, 2019). We calculated all estimates using random-effects and consistency models if the node-split method (Dias et al., 2010) determined that the direct effect was consistent with the indirect effect. In contrast, the inconsistency model would be utilized to estimate the results (Dias et al., 2010). We set the following parameters to run calculation of estimates: 1) four chains, 2) 20,000 tuning iterations and 50,000 simulation iterations, 3) a thinning interval of 10, 4) 10,000 inference samples, and 5) a variance scaling factor of 2.5. We used OR or SMD with a 95% creditable interval (CrI) to express all estimates of network meta-analysis. We evaluated the convergence of data using the Brooks Gelman–Rubin statistical method and reliable convergence was thought to be achieved if the potential proportional reduction factor (PRF) was close to 1 (Brooks and Gelman, 1998; Burger and Schall, 2015). We calculated the surface under the cumulative ranking curve to rank all intravenous GC regimes (Salanti et al., 2011). Stata 14.0 was utilized to generate the comparison-adjusted funnel plot for the purpose of qualitatively inspecting whether the presence of publication bias when the accumulated number of eligible studies for individual comparison was more than 10 (Palma Perez and Delgado Rodriguez, 2006), and an asymmetric funnel plot suggested the presence of publication bias (Page et al., 2018). Moreover, we used Microsoft Excel to graphically depict the results of the network meta-analysis and generate the ranking plot according to the original data calculated using the ADDIS software.

## Results

### Identification and Selection of Studies

**FIGURE 1:**
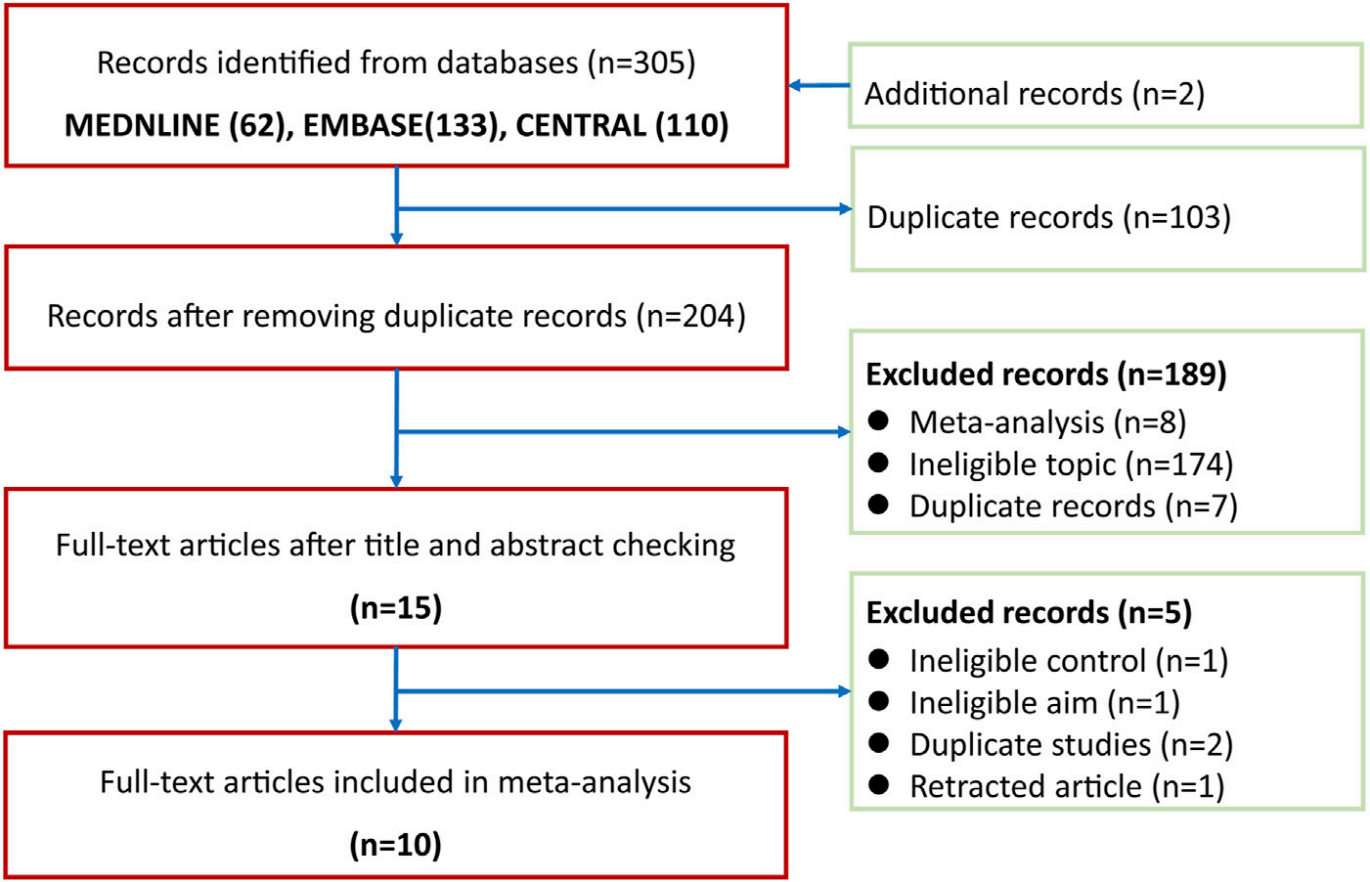
Flow chart of identification and selection of studies. CENTRAL, the Cochrane Central Register of Controlled Trials.

A total of 305 records were identified after electronically searching Medline (*n* = 62), EMBASE (*n* = 133), and CENTRAL (*n* = 110). All records were downloaded from databases and were then imported into EndNote X9. A total of 103 duplicates were removed by the function of locating duplication of the EndNote software. We retrieved full-texts of 15 records after excluding 189 ineligible records which were excluded based on the screening of titles and abstracts. A total of 10 eligible studies (Macchia et al., 2001; Marcocci et al., 2001; Kauppinen-Mäkelin et al., 2002; Kahaly et al., 2005; Aktaran et al., 2007; Akarsu et al., 2011; Zhu et al., 2014; Roy et al., 2015; He et al., 2017; Mu et al., 2020) were included into the final meta-analysis after excluding five ineligible studies due to four following reasons: 1) ineligible control regime (*n* = 1), ineligible aim (*n* = 1), duplicate studies (*n* = 2), and retracted study (*n* = 1). The process of identifying and selecting eligible studies is presented in Figure 1.

### Characteristics of 10 Eligible Studies

All eligible studies have been published between 2001 and 2020. Among 10 eligible studies, three studies (Zhu et al., 2014; He et al., 2017; Mu et al., 2020) were conducted in China, two studies were conducted in Turkey (Aktaran et al., 2007; Akarsu et al., 2011) and Italy (Macchia et al., 2001; Marcocci et al., 2001), and the remaining three studies were conducted in Germany (Kahaly et al., 2005), Finland (Kauppinen-Mäkelin et al., 2002), and India (Roy et al., 2015). The sample size of the individual study was between 33 and 90, with a medium number of 57 and a cumulative number of 593. Eight studies (Marcocci et al., 2001; Kahaly et al., 2005; Aktaran et al., 2007; Akarsu et al., 2011; Zhu et al., 2014; Roy et al., 2015; He et al., 2017; Mu et al., 2020) enrolled patients with moderate-to-severe GO, and the remaining two studies (Macchia et al., 2001; Kauppinen-Mäkelin et al., 2002) enrolled patients with mild-to-moderate GO. The details of the 10 eligible studies are presented in Table 1.

**TABLE 1:** Basic characteristics of all included studies in this network meta-analysis.

References	Country	Sample size (male)	Mean age, yrs	Disease severity	GO duration (mos)	Details of interventions		Follow-up
Akarsu et al. (2011)	Turkey	18 (7) vs. 15 (5)	28.4 vs. 29.6	Moderately severe active GO	3.1 vs. 2.9	0.5 g iv MP for 6 wks, followed by 0.25 g weekly for 6 wks (WR)	72 mg po MP for 2 wks, followed by 8 mg for 2 wks (OGC)	24 wks
Aktaran et al. (2007)	Turkey	25 (11) vs. 27 (13)	44.3 vs. 41.3	Moderately severe active GO	1–5	0.5 g iv MP for 6 wks, followed by 0.25 g weekly for 6 wks (WR)	72 mg po MP for 2 wks, followed by 8 mg for 2 wks (OGC)	3 mos
He et al. (2017)	China	22 (6) vs. 18 (8)	42.3 vs. 41.2	Moderately severe GO	7 vs. 6	1.5 g iv MP monthly for 3 mos (MR)	0.5 g iv MP weekly for 6 wks, followed by 0.25 g for 6 wks (WR)	13 wks
Kahaly et al. (2005)	Germany	35 (10) vs. 35 (11)	52 vs. 48	Moderately severe GO	4 vs. 3	0.5 g iv MP for 6 wks, followed by 0.25 g weekly for 6 wks (WR)	0.1 g po PS for 2 wks, followed by 0.01 g for 2 wks (OGC)	6 mos
Kauppinen-Mäkelin et al. (2002)	Finland	18 (1) vs. 15 (1)	46.4 vs. 46.1	Mildly moderate GO	n.r.	total 4.16 g MP weekly for 16 wks (WR)	total 2.99 g PS for 16 wks (OGC)	12 mos
Macchia et al. (2001)	Italy	25 (6) vs. 26 (5)	42.6 vs. 44.6	Mildly moderate GO	n.r.	1 g iv MP weekly for 6 wks (WR)	60–80 mg po PS for 6 wks (OGC)	1 yr
Marcocci et al. (2001)	Italy	41 (6) vs. 41 (8)	50 vs. 48	Moderately severe GO	35 vs. 34	total 9–12 g iv MP weekly for 14 wks (WR)	total 2.99 g PS for 14 wks (OGC)	2 mos
Mu et al. (2020)	China	46 (18) vs. 44 (20)	35.2 vs. 34.8	Moderately severe active TAO	12.6 vs. 6.6	0.5 g iv MP for 6 wks, followed by 0.25 g weekly for 6 wks (WR)	0.5 g iv MP daily for 5 days, followed by po MP for 3 mos (DR)	12 wks
Roy et al. (2015)	India	31 (9) vs. 31 (15)	37.6 vs. 36.9	Moderately severe active GO	n.r.	0.5 g iv MP monthly for 4 mos (MR)	1 mg po PS for 6 wks then tapered stopped (OGC)	12 mos
Zhu et al. (2014)	China	39 (15) vs. 41 (19)	45.3 vs. 48.2	Moderately severe active GO	13.6 vs. 6.4	0.5 g iv MP for 6 wks, followed by 0.25 g weekly for 6 wks (WR)	0.5 g iv MP daily for 2 wks, followed by 0.25 g for 2 wks and then by tapering po PS (DR)	12 wks

GO, Graves’ ophthalmopathy; TAO, thyroid-associated ophthalmopathy; DR, daily regime; WR, weekly regime; MR, monthly regime; OGC, oral glucocorticoids; yrs, years; mos, months; wks, weeks; iv, intravenous; po, oral; MP, methylprednisolone; PS, prednisolone; n.r., not reported.

### Risk of Bias

**FIGURE 2:**
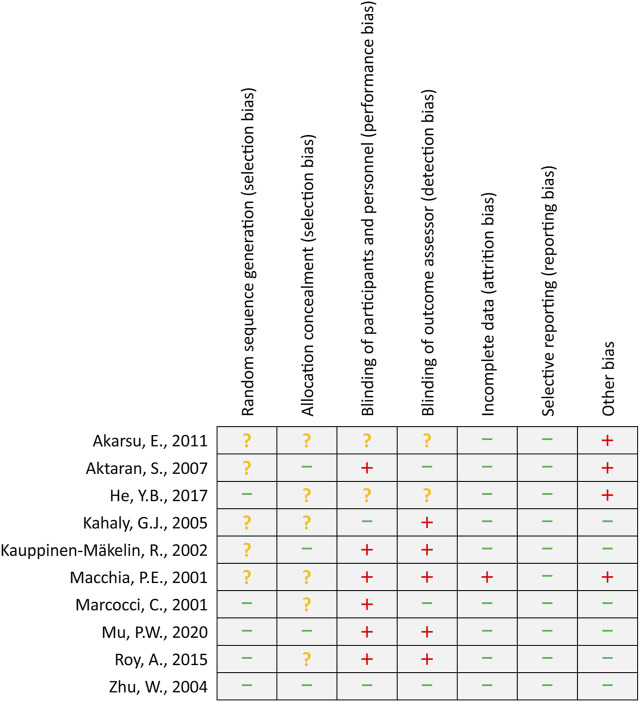
Risk of bias summary. The green minus sign, yellow question mark, and red plus sign represent low, unclear, and high risk of bias, respectively.

A total of five studies (Macchia et al., 2001; Zhu et al., 2014; Roy et al., 2015; He et al., 2017; Mu et al., 2020) reported the details of generating a random sequence, and four studies (Kauppinen-Mäkelin et al., 2002; Aktaran et al., 2007; Zhu et al., 2014; Mu et al., 2020) clearly introduced the methods of allocation concealment. Two (Kahaly et al., 2005; Zhu et al., 2014) and three (Marcocci et al., 2001; Aktaran et al., 2007; Zhu et al., 2014) studies appropriately avoided performance bias and detection bias, respectively. Nine studies (Marcocci et al., 2001; Kauppinen-Mäkelin et al., 2002; Kahaly et al., 2005; Aktaran et al., 2007; Akarsu et al., 2011; Zhu et al., 2014; Roy et al., 2015; He et al., 2017; Mu et al., 2020) reported complete data or used appropriate statistical methods to process results. All studies reported anticipated outcomes. Four studies (Macchia et al., 2001; Aktaran et al., 2007; Akarsu et al., 2011; He et al., 2017) were labeled with a high risk of bias due to their small sample size or the design of the pilot study. The details of the risk of bias assessment are summarized in Figure 2.

### Structure of Available Evidence

**FIGURE 3:**
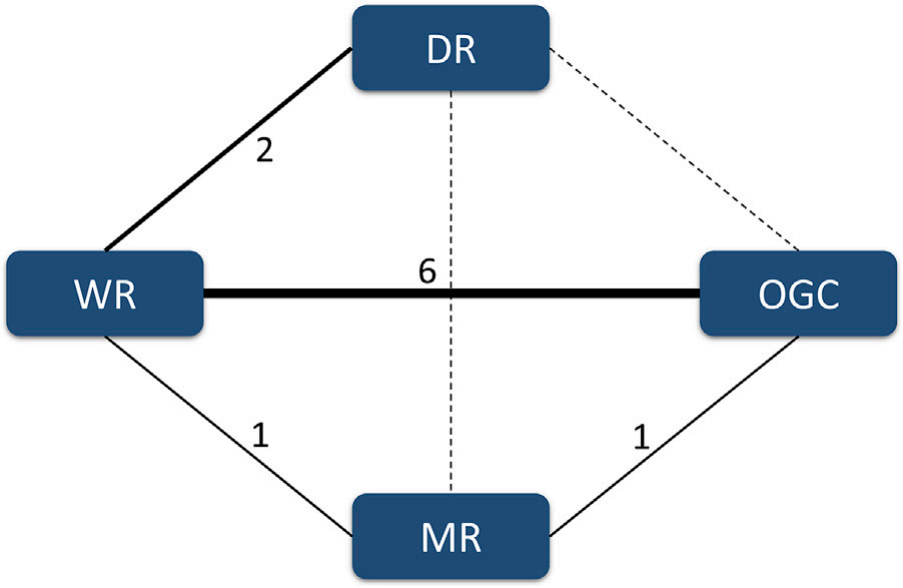
Evidence structure of available comparisons. The solid line indicates the presence of a direct comparison of two regimes, and the dotted line suggests the absence of a direct comparison between two regimes. The width of the solid line is positively related to the accumulated number of eligible studies.

In the present network meta-analysis, we identified four comparisons involving four regimes. More specifically, six studies (Macchia et al., 2001; Marcocci et al., 2001; Kauppinen-Mäkelin et al., 2002; Kahaly et al., 2005; Aktaran et al., 2007; Akarsu et al., 2011) reported the comparison between the WR and OGC regime, two studies (Zhu et al., 2014; Mu et al., 2020) reported the comparison between the WR and DR, one study (He et al., 2017) reported the comparison between the WR and MR, and the remaining one study (Roy et al., 2015) reported the comparison between the MR and OGC regime. The structure of available evidence is illustrated in Figure 3.

### Reporting Bias and Inconsistency

The accumulated number of eligible studies for an individual outcome was lower than 10, and thus, we did not generate a comparison-adjusted funnel plot to detect the possibility of reporting bias. Moreover, a first-level closed loop was available for two outcomes, namely, response and CASC, and the results indicated the absence of inconsistency between direct and indirect effects. The results of the inconsistency test are summarized in Supplementary Table S2.

### Meta-Analysis of Response

**FIGURE 4:**
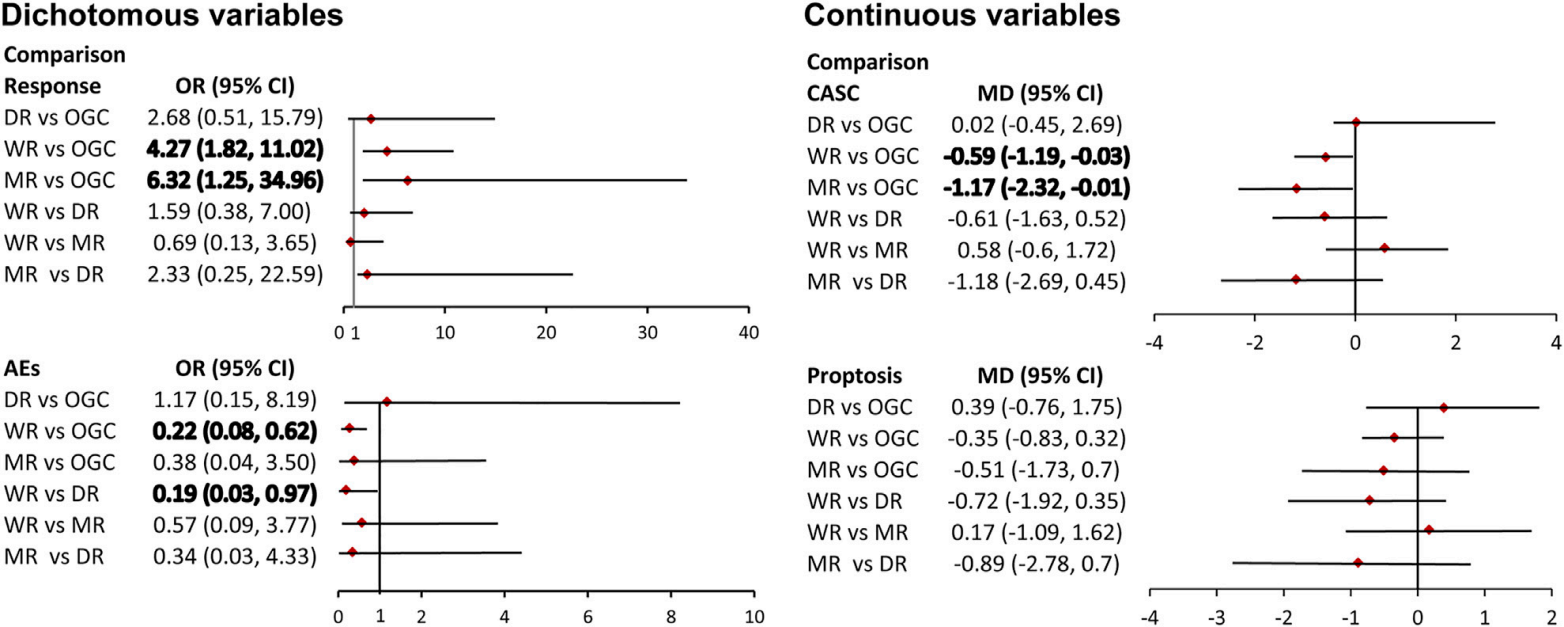
Pooled results of network meta-analysis. The bold number indicates a significant difference.

Four direct comparisons were available for response, namely, the comparison between the WR and OGC regime, comparison between the MR and OGC regime, the comparison between the WR and DR, and comparison between the WR and MR. Pooled results from direct meta-analysis suggested that the WR (six studies; OR, 3.85; 95% CI; 2.28–6.49) and MR (1 study; OR, 5.56; 95% CI; 1.57–19.72) were superior to OGC regime (Supplementary Figure S1), which were supported by network meta-analysis (WR vs. OGC: OR, 4.27; 95% CrI; 1.82 to 11.02; MR vs. OGC: OR, 6.32; 95% CrI; 1.25–34.96) (Figure 4).

### Meta-Analysis of Clinical Activity Score Change

Four direct comparisons were available for CASC, namely, the comparison between the WR and OGC regime, the comparison between the MR and OGC regime, the comparison between the WR and DR, and comparison between the WR and MR. Pooled results from pairwise meta-analysis suggested that the WR (six studies; MD, −0.54; 95% CI; −1.00 to −0.09) and MR (one study; MD, −1.55; 95% CI; -2.18 to −0.92) were superior to the OGC regime (Supplementary Figure S1), which were supported by network meta-analysis (WR vs. OGC: SMD, -0.59; 95% CrI; -1.19 to 1–0.03; MR vs. OGC: SMD, -1.17; 95% CrI; −2.32 to −0.01) (Figure 4).

### Meta-Analysis of Proptosis

Three direct comparisons were available for proptosis, namely, the comparison between the WR and OGC regime, the comparison between the MR and OGC regime, and the comparison between the WR and DR. Pooled results of the direct meta-analysis suggested no statistical difference in the three comparisons (Supplementary Figure S1), which were consistent with the results of network meta-analysis (Figure 4).

### Meta-Analysis of Adverse Events

Three direct comparisons were available for AEs, namely, the comparison between the WR and OGC regime, the comparison between the WR and DR, and the comparison between the WR and MR. Pooled results from pairwise meta-analysis suggested that the WR was associated with a lower risk of AEs than the OGC regime (3 studies; OR, 0.23; 95% CI; 0.12–0.44) and the DR (a study; OR, 0.20; 95% CI; 0.08–0.50) (Supplementary Figure S1), which were supported by network meta-analysis (WR vs. OGC: OR, 0.22; 95% CrI; 0.08 to 0.62; WR vs. DR: OR, 0.19; 95% CrI; 0.03–30.97) (Figure 4).

### Ranking of Three Intravenous Glucocorticoid Regimes

We calculated ranking probabilities of different intravenous GC regimes in terms of all outcomes, including response, CASC, proptosis, and AEs, and all results are graphically depicted in Supplementary Figure S2. According to the results of ranking probabilities, the MR has the highest probability of becoming the best treatment option in terms of response (65.0%), followed by the WR (61.0%), DR (60.0%), and OGC regime (89.0%); the DR has the highest probability of becoming the best regime in terms of CASC (83.0%), followed by the WR (77.0%), OGC regime (50.0%), and DR (51.0%); the MR has the highest probability of becoming the best regime in terms of proptosis (63.0%), followed by the WR (56.0%), OGC regime (60.0%), and DR (72.0%); the WR has the highest probability of becoming the best regime in terms of AEs (72.0%), followed by the MR (53.0%), OGC regime (51.0%), and DR (55.0%).

## Discussion

GO remains a critically important clinical problem around the world and requires positive therapy (Bahn, 2010). Although several treatment regimens, such as glucocorticoid therapy and decompression surgery, have been proposed for the treatment of GO, glucocorticoid therapy has been recommended as the first-line option by recognized guidelines (Bartalena et al., 2008). Previous meta-analyses have established that intravenous GC therapy was superior to oral GC therapy, and one meta-analysis also investigated the comparative efficacy and safety of different doses of intravenous GC therapy; however, it is unclear which frequencies of intravenous administration of GC therapy should be preferably selected in the real settings.

As we know, this has been the first network meta-analysis of determining the optimal frequency of intravenous GC therapy to date. Our network meta-analysis suggested that the WR and MR significantly increased the response when compared with the OGC regime. So far, six studies (Macchia et al., 2001; Marcocci et al., 2001; Kauppinen-Mäkelin et al., 2002; Kahaly et al., 2005; Aktaran et al., 2007; Akarsu et al., 2011) have investigated the comparative response between the WR and OGC regime, and three studies (Macchia et al., 2001; Aktaran et al., 2007; Akarsu et al., 2011) with an extremely small sample size did not detect a significant difference; three other studies (Marcocci et al., 2001; Kauppinen-Mäkelin et al., 2002; Kahaly et al., 2005) with a relatively larger sample size detected a statistical difference between the WR and OGC regime in terms of response. Moreover, one study (Macchia et al., 2001) enrolled patients with mildly moderate GO, which is more sensitive to treatments. Only one study (Roy et al., 2015) which enrolled 62 patients compared the MR with OGC regime and found that the MR was superior to the OGC regime in terms of response, which was consistent with our finding. An accumulated sample size of 321 was obtained through pooling results from these six studies, and thus, a more reliable and robust result was generated. Meanwhile, we also determined that the MR and WR have a relatively higher possibility of becoming the preferred treatment option based on accumulated data.

The same studies introduced above also reported CASC when comparing the WR (Macchia et al., 2001; Marcocci et al., 2001; Kauppinen-Mäkelin et al., 2002; Kahaly et al., 2005; Aktaran et al., 2007; Akarsu et al., 2011) and MR (Roy et al., 2015) with OGC. Four studies (Macchia et al., 2001; Marcocci et al., 2001; Kahaly et al., 2005; Aktaran et al., 2007) supported that the MR significantly reduced CAS compared with OGC, which was consistent with our result. However, the remaining two studies (Kauppinen-Mäkelin et al., 2002; Akarsu et al., 2011) reported results inconsistent with our findings. It must be noted that the sample size of the two studies with inconsistent conclusions was extremely small, and thus, the reliability and robustness of their findings will be greatly compromised. Compared with those two studies, our network meta-analysis accumulated more sample size, and thus, more trustworthy results were generated. Similarly, we determined the MR and WR to have a relatively higher possibility of being the preferred treatment option in terms of CASC.

A total of three studies (Marcocci et al., 2001; Kahaly et al., 2005; Akarsu et al., 2011), compared the WR with the OGC regime, and two studies (Marcocci et al., 2001; Kahaly et al., 2005) found a statistical difference between these two regimes, which was consistent with our result. However, one other study (Akarsu et al., 2011) generated a conflicting result. This is not surprising as 33 patients were enrolled in that study, which reported an inconsistent result (Akarsu et al., 2011). By contrast, a total of 185 patients were enrolled in our network meta-analysis to generate more reliable results. Only one study (Mu et al., 2020) reported a direct comparison between the WR and DR for AEs and found that the WR was associated with a lower risk of AEs, which was consistent with our finding. However, no additional direct evidence has been provided to increase the power of our network meta-analysis, and therefore, more studies are suggested to explore this issue.

Regardless of the fact that the present network meta-analysis generated several interesting findings due to several strengths, some limitations must be acknowledged. First and foremost, only 10 eligible studies with a limited sample size were included in the final analysis, which impairs the robustness of pooled results very possibly. Second, two studies enrolled patients with mildly moderate GO; however, subgroup analysis and sensitivity analysis were not performed due to limited data. Third, 2 GCs including MP and PS were prescribed in original studies; however, we did not separately investigate the efficacy and safety of two different GCs. Fourth, the duration of GO varied from one study to another, and subgroup analysis was not conducted due to limited data. Fifth, variations in dose in all eligible studies cannot be ignored, and no subgroup analysis can be designed owing to the limited number of eligible studies. As a result, pooled results may be impaired because the previous meta-analysis has established the dose–response relationship of GC therapy (Zang et al., 2011). Sixth, no study has been conducted to directly compare the MR with the DR, and comparative efficacy and safety between these two regimes were solely obtained based on indirect evidence. Hence, this result should be cautiously interpreted when one wants to apply our findings in clinical settings.

## Conclusion

Based on the best available evidence, we conclude that the WR or MR should be preferentially prescribed to treat moderate-to-severe GO because the WR or MR is significantly associated with improved response, reduced CAS, and lower AEs than the OGC regime. However, more studies with a large sample size should be conducted to further confirm our findings and compare the WR with the MR. Moreover, we also suggest developing further studies that directly investigate the comparative effects between the MR and DR because no direct comparison has been available to date.

## Data Availability

The original contributions presented in the study are included in the article/Supplementary Material, further inquiries can be directed to the corresponding author.

## Supplementary Material

Supplementary Figure S1 Pooled results of direct meta-analysis. CASC, clinical activity score change; AEs, adverse events; OR, odds ratio; MD, mean difference.

Supplementary Figure S2 Rank probability of all studied treatment regimes for each outcome. CASC, clinical activity score change; AEs, adverse events.

Supplementary Table S1 Search strategy of target databases.

Supplementary Table S2 Inconsistency examination for response and CASC based on the node split method. CASC, clinical activity score change.
