# Supplementary material for: Network Meta-Analysis of Different Intravenous Glucocorticoid Regimes for the Treatment of Graves’ Orbitopathy
Source: Front Pharmacol. 2022 Apr 26;13:785757. doi: 10.3389/fphar.2022.785757 (PMC9086427; doi:10.3389/fphar.2022.785757)
Supplement: Supplementary file 1 [file Table1.DOCX]

1. **Search strategy of Medline using PubMed**

| **NO.** | **Search query** | **Results** |
| --- | --- | --- |
| 1 | (("Glucocorticoids"[Mesh]) OR "Methylprednisolone"[Mesh]) OR "Steroids"[Mesh] | 905,632 |
| 2 | ((((((Glucocorticoid[Title/Abstract]) OR (Glucocorticoids[Title/Abstract])) OR (Glucocorticoid Effect[Title/Abstract])) OR (Glucocorticoid Effects[Title/Abstract])) OR (Methylprednisolone[Title/Abstract])) OR (Steroid[Title/Abstract])) OR (Steroids[Title/Abstract]) | 280,722 |
| 3 | #1 OR #2 | 1,037,406 |
| 4 | "Graves Ophthalmopathy"[Mesh] | 2,355 |
| 5 | ((((((((((((((((Graves Ophthalmopathy[Title/Abstract]) OR (Thyroid-Associated Ophthalmopathies[Title/Abstract])) OR (Thyroid Associated Ophthalmopathies[Title/Abstract])) OR (Dysthyroid Ophthalmopathy[Title/Abstract])) OR (Dysthyroid Ophthalmopathies[Title/Abstract])) OR (Thyroid-Associated Ophthalmopathy[Title/Abstract])) OR (Thyroid Associated Ophthalmopathy[Title/Abstract])) OR (Graves Orbitopathy[Title/Abstract])) OR (Graves Orbitopathies[Title/Abstract])) OR (Myopathic Ophthalmopathy[Title/Abstract])) OR (Myopathic Ophthalmopathies[Title/Abstract])) OR (Congestive Ophthalmopathy[Title/Abstract])) OR (Congestive Ophthalmopathies[Title/Abstract])) OR (Edematous Ophthalmopathy[Title/Abstract])) OR (Edematous Ophthalmopathies[Title/Abstract])) OR (Infiltrative Ophthalmopathies[Title/Abstract])) OR (Infiltrative Ophthalmopathy[Title/Abstract]) | 3,494 |
| 6 | #4 OR #5 | 1,403 |
| 7 | ("Randomized Controlled Trial" [Publication Type]) OR "Randomized Controlled Trials as Topic"[Mesh] | 678,773 |
| 8 | random*[Title/Abstract] | 1,234,617 |
| 9 | #7 OR #8 | 1,418,222 |
| 10 | #3 AND #6 AND #9 | 62 |

1. **Search strategy of Embase using OVID**

| **NO.** | **Search query** | **Results** |
| --- | --- | --- |
| 1 | (Glucocorticoids or Glucocorticoid Effect or Glucocorticoid Effects or Methylprednisolone or Steroid or Steroids).af. | 531640 |
| 2 | (Graves Ophthalmopathy or Thyroid-Associated Ophthalmopathies or Thyroid Associated Ophthalmopathies or Dysthyroid Ophthalmopathy or Dysthyroid Ophthalmopathies or Thyroid-Associated Ophthalmopathy or Thyroid Associated Ophthalmopathy or Graves Orbitopathy or Graves Orbitopathies or Myopathic Ophthalmopathy or Myopathic Ophthalmopathies or Congestive Ophthalmopathy or Congestive Ophthalmopathies or Edematous Ophthalmopathy or Edematous Ophthalmopathies or Infiltrative Ophthalmopathies or Infiltrative Ophthalmopathy).af. | 4618 |
| 3 | random*.af. | 1913204 |
| 4 | (Glucocorticoids or Methylprednisolone or Steroids).sh. | 101625 |
| 5 | 1 or 4 | 531640 |
| 6 | Graves Ophthalmopathy.sh. | 69 |
| 7 | 2 or 6 | 4618 |
| 8 | 3 and 5 and 7 | 133 |

1. **Search strategy of CENTRAL using OVID**

| **NO.** | **Search query** | **Results** |
| --- | --- | --- |
| 1 | (Glucocorticoid or Glucocorticoids or Glucocorticoid Effect or Glucocorticoid Effects or Methylprednisolone or Steroid or Steroids).af. | 33637 |
| 2 | (Glucocorticoids or Methylprednisolone or Steroids).sh. | 7392 |
| 3 | 1 or 2 | 33637 |
| 4 | (Graves Ophthalmopathy or Thyroid-Associated Ophthalmopathies or Thyroid Associated Ophthalmopathies or Dysthyroid Ophthalmopathy or Dysthyroid Ophthalmopathies or Thyroid-Associated Ophthalmopathy or Thyroid Associated Ophthalmopathy or Graves Orbitopathy or Graves Orbitopathies or Myopathic Ophthalmopathy or Myopathic Ophthalmopathies or Congestive Ophthalmopathy or Congestive Ophthalmopathies or Edematous Ophthalmopathy or Edematous Ophthalmopathies or Infiltrative Ophthalmopathies).af. | 329 |
| 5 | Graves Ophthalmopathy.sh. | 106 |
| 6 | 4 or 5 | 329 |
| 7 | random*.af. | 1424247 |
| 8 | Randomized Controlled Trial.pt. or Randomized Controlled Trials as Topic.sh. | 521545 |
| 9 | 7 or 8 | 1424247 |
| 10 | 3 and 6 and 9 | 110 |

CENTRAL, Cochrane central register of controlled trials.
