# Supplementary figures and images for: Network Meta-Analysis of Different Intravenous Glucocorticoid Regimes for the Treatment of Graves’ Orbitopathy
Source: Front Pharmacol. 2022 Apr 26;13:785757. doi: 10.3389/fphar.2022.785757 (PMC9086427; doi:10.3389/fphar.2022.785757)

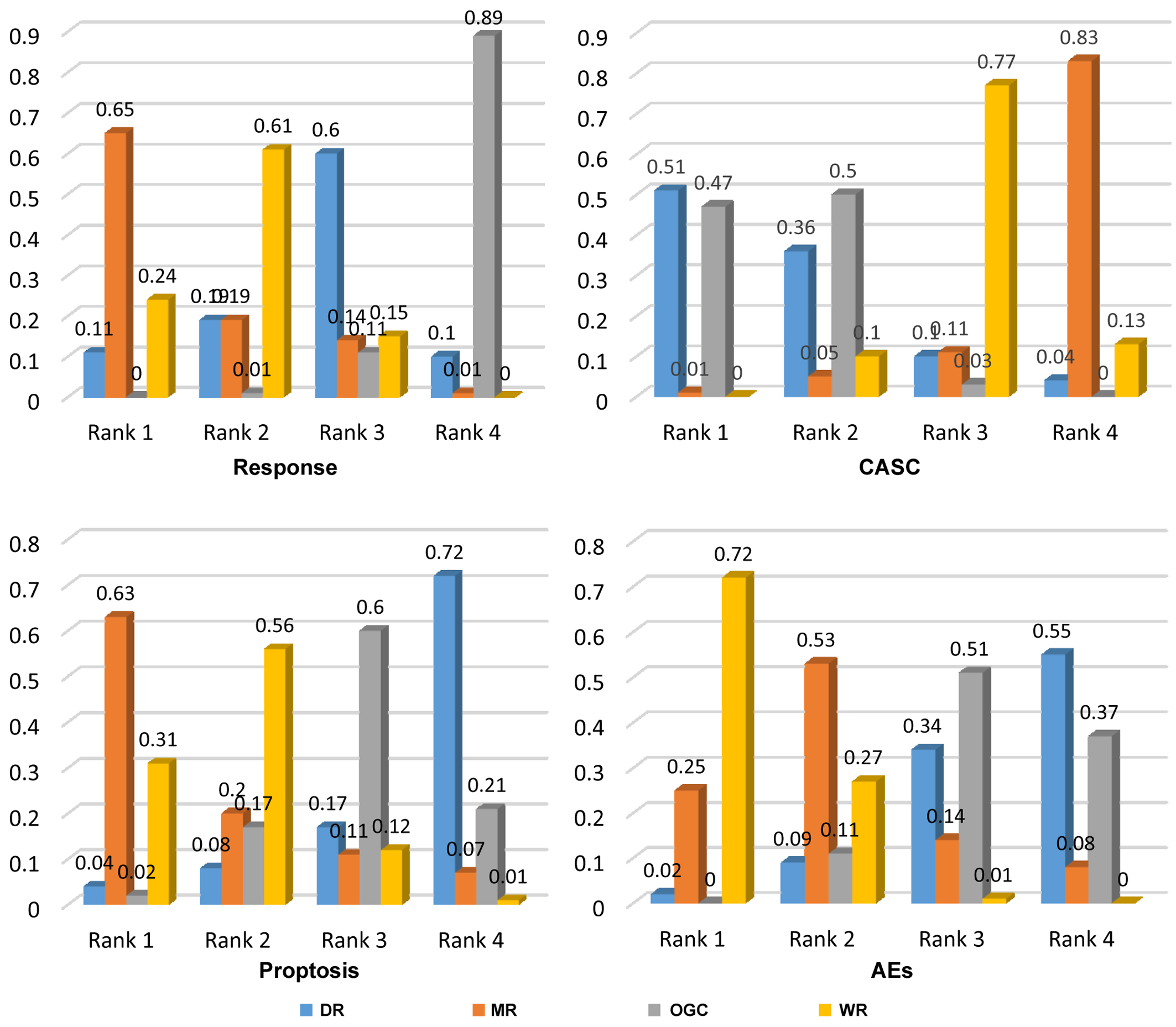

Supplement: Supplementary file 2 [file Image2.TIF]

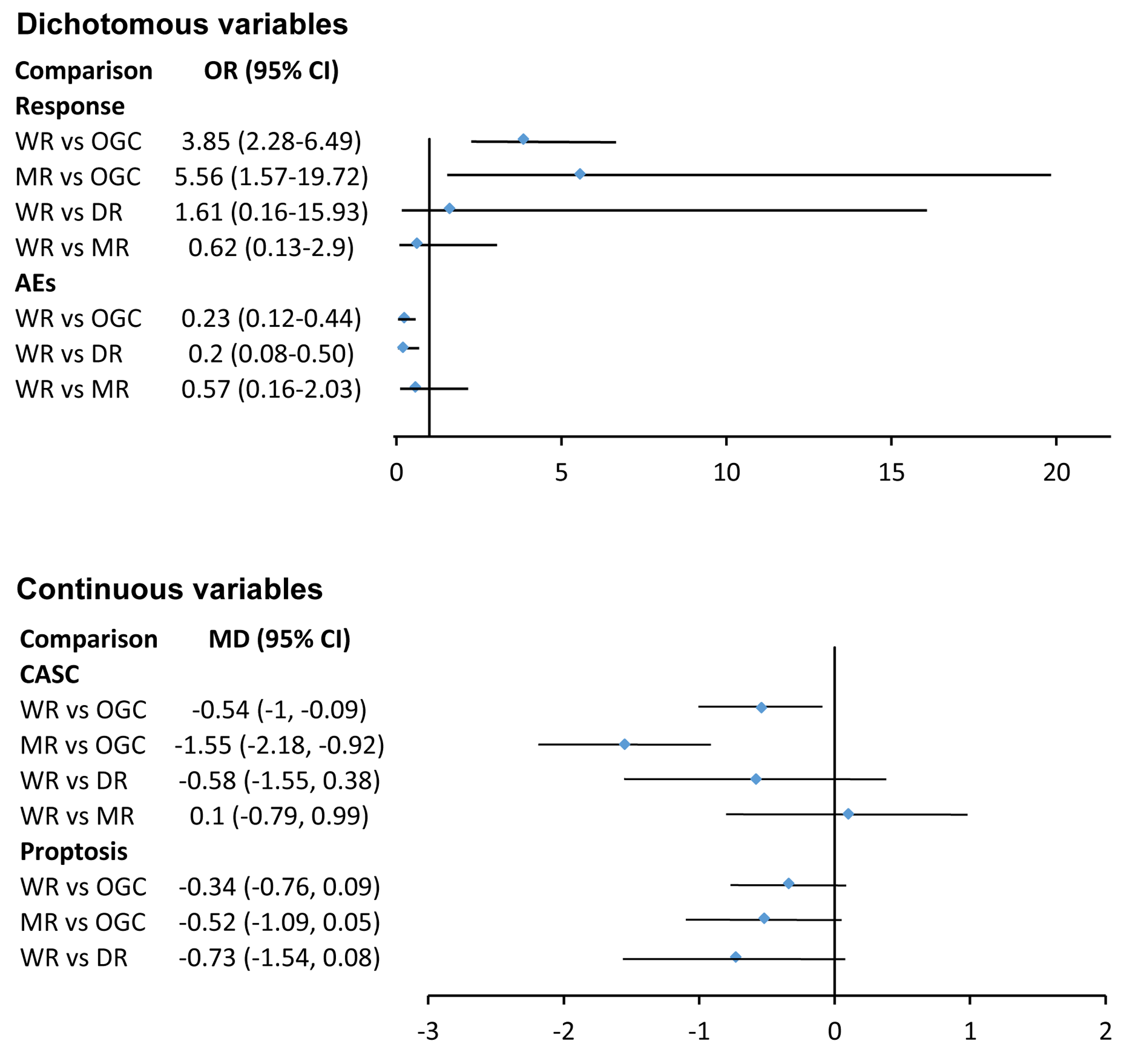

Supplement: Supplementary file 3 [file Image1.TIF]
