# Supplementary material for: Network Meta-Analysis of Different Intravenous Glucocorticoid Regimes for the Treatment of Graves’ Orbitopathy
Source: Front Pharmacol. 2022 Apr 26;13:785757. doi: 10.3389/fphar.2022.785757 (PMC9086427; doi:10.3389/fphar.2022.785757)
Supplement: Supplementary file 4 [file Table2.DOCX]

**Inconsistency test for response**

| **Name** | **Direct Effect** | **Indirect Effect** | **Overall** | **P-Value** |
| --- | --- | --- | --- | --- |
| MR-OGC | -1.80 (-4.25, 0.57) | -1.98 (-4.76, 0.74) | -1.84 (-3.55, -0.22) | 0.90 |
| MR-WR | -0.50 (-3.13, 2.11) | -0.33 (-2.93, 2.42) | -0.38 (-2.08, 1.29) | 0.93 |
| OGC-WR | 1.48 (0.51, 2.53) | 1.31 (-2.10, 4.85) | 1.45 (0.60, 2.40) | 0.91 |

**Inconsistency test for CASC**

| **Name** | **Direct Effect** | **Indirect Effect** | **Overall** | **P-Value** |
| --- | --- | --- | --- | --- |
| MR, OGC | 1.56 (-0.01, 3.11) | 0.64 (-1.20, 2.44) | 1.17 (0.01, 2.32) | 0.41 |
| MR, WR | 0.10 (-1.56, 1.79) | 1.00 (-0.74, 2.70) | 0.58 (-0.60, 1.72) | 0.42 |
| OGC, WR | -0.54 (-1.20, 0.05) | -1.44 (-3.72, 0.87) | -0.59 (-1.19, -0.03) | 0.41 |

CASC, clinical activity score change.

In the present network meta-analysis, first closed loop was available for two outcomes including response and CASC. We therefore utilized node split method to perform inconsistency test and a P value of more than 0.05 indicates absence of inconsistency between direct and indirect effects.
